# Descriptive epidemiology and outcomes of bone sarcomas in adolescent and young adult patients in Japan

**DOI:** 10.1186/s12891-018-2217-1

**Published:** 2018-08-18

**Authors:** Takashi Fukushima, Koichi Ogura, Toru Akiyama, Katsushi Takeshita, Akira Kawai

**Affiliations:** 1 Department of Orthopaedic Surgery, Saitama Medical Center, Jichi Medical University, Saitama, Japan; 2 Department of Orthopaedic Surgery, Jichi Medical University, Tochigi, Japan; 3 Department of Musculoskeletal Oncology, National Cancer Center Hospital, Tokyo, Japan; 4 Department of Orthopaedic Surgery, Faculty of Medicine, The University of Tokyo, Tokyo, Japan

**Keywords:** Bone sarcoma, Adolescent and young adult, Cancer survival, Japan, Database

## Abstract

**Background:**

There have been fewer improvements in the clinical outcomes of adolescent and young adult (AYA) patients with cancer than for children and older adults, possibly because fewer studies focus on patients in this age group. The aims of this study were (1) to determine survival rates of bone sarcoma among AYAs in Japan (for comparison with other age groups), and (2) to establish whether belonging to the AYA age group at diagnosis was correlated with poor cancer survival in Japan.

**Methods:**

A total of 3457 patients diagnosed with bone sarcoma (1930 male and 1527 female) were identified from 63,931 records in the Bone and Soft Tissue Tumor (BSTT) registry, a nationwide Japanese database, from 2006 to 2013. The histologic subtypes of bone sarcoma were osteosarcoma, chondrosarcoma, and Ewing sarcoma. The primary endpoints for prognosis were the occurrence of tumor-related death. We compared the epidemiological features of AYAs with other age groups. The cancer survival rates were calculated using the Kaplan-Meier method. Cox proportional hazards models were used to analyze the prognostic factors for cancer survival.

**Results:**

The majority of AYA had osteosarcoma 631 (56.2%), while 198 (17.6%) had chondrosarcoma. The frequency of bone sarcoma occurrence was highest among AYA patients, who accounted for a marked proportion of patients with each type of sarcoma. With the exception of sarcoma type, AYA patients did not significantly differ from patients in other age groups for any of the investigated clinicopathological parameters. Cancer survival of AYA patients was significantly higher than in the elderly. Univariate and multivariate analyses revealed that AYA status was not a predictor of poor cancer survival. However, older age (≥65 years) was a predictor of poor cancer survival in patients with overall bone sarcoma, osteosarcoma, chondrosarcoma.

**Conclusion:**

This epidemiological study is the first to investigate AYA patients with bone sarcoma using the nationwide BSTT Registry. We found that cancer survival of AYA patients was significantly higher than that of the elderly. AYA status was not a predictor of poor cancer survival in Japan.

**Electronic supplementary material:**

The online version of this article (10.1186/s12891-018-2217-1) contains supplementary material, which is available to authorized users.

## Background

There have been significant advances in the early detection and treatment of cancer, which have led to improvements in overall survival rates in general patient populations over several decades [1]. However, the clinical outcomes of adolescent and young adult (AYA) patients, defined as those between the ages of 15 to 39, with cancer have not improved [1–4]. One explanation for this is that, to date, little attention and few resources have been devoted to studying the incidence, biology, and treatment outcomes in AYA patients with cancer [5].

AYA patients with cancer are predominantly afflicted by lymphoma, melanoma, testicular cancer, sarcoma, thyroid cancer, leukemia, and breast cancer [5]. Sarcomas comprise up to 6% of total malignancies in AYAs and represent one of the most common types of cancer in this population [5]. However, sarcoma is generally a rare disease, and its estimated total crude incidence rate in Europe is 5.6 per 100,000 individuals per year [6]. A few previous studies have investigated the clinical outcomes of AYAs with bone sarcoma using nationwide or large databases with sufficient numbers of patients. However, most previous studies were based on data derived from small numbers of cases, and those with larger sample sizes have only analyzed a few disease-related factors [7–11].

In Japan, no studies on the epidemiology and clinical outcomes of AYA patients with sarcoma compared with patients diagnosed at other ages have been conducted because of the lack of a suitable database. In 2014, the Bone and Soft Tissue Tumor (BSTT) registry—a nationwide organ-specific cancer registry for bone and soft tissue tumors in Japan—became available for the purposes of clinical research, enabling a large-scale nationwide epidemiological investigation of AYA patients with sarcoma.

The aims of the present study were: 1) to determine survival rates of bone sarcoma among AYAs in Japan (for comparison with other age groups), and 2) to establish whether belonging to the AYA age group at diagnosis was correlated with poor cancer survival in Japan.

## Methods

### Data source

The BSTT Registry is a nationwide patient data collection system for organ-specific bone and soft tissue tumors that was launched in the 1950s by the Japanese Orthopaedic Association (JOA). All JOA-certified hospitals of musculoskeletal oncology (89 facilities) are required to participate in the registry; hence, almost all musculoskeletal malignant tumor cases treated by Japanese orthopedic surgeons are registered.

Detailed data of patients with primary bone and soft tissue tumors (both benign and malignant) and metastatic bone tumors treated at the participating hospitals are collected annually. The BSTT registry survey of patients diagnosed from January 1 to December 31 of the previous year are conducted annually in May. The survey includes basic demographic data of the patient, as well as information on the tumor, surgery, and treatment other than surgery. The next survey is conducted 2, 5, and 10 years after the initial registration at prognosis. The data for patients with bone and soft tissue sarcomas (not for patients with benign and metastatic bone tumors) are collected. It includes information on several outcomes at the time of the latest follow-up.

The BSTT Registry is similar to the Surveillance, Epidemiology, and End Results Program database in the United States; however, it has some additional advantages in that data are provided by the treating physicians themselves, and include histologic findings, treatment modalities, and surgical, functional, and oncologic outcomes. The Musculoskeletal Tumor Committee of the JOA approved the use of the BSTT Registry for the purposes of clinical research in 2014 [12].

Study approval was obtained from the Institutional Review Board of the JOA.

### Data extraction

The focus of this study was only bone sarcomas recorded in the BSTT Registry for patients diagnosed between 2006 and 2013. Data on 3457 patients with primary bone sarcoma were extracted from the database that encompassed 63,931 patients. Of these, 521, 1123, 982, and 831 were patients aged ≤14 years (children), 15–39 years (AYAs), 40–64 years (adults), and ≥ 65 years (elderly), respectively. The analyzed data included the year of registration; demographic characteristics; tumor size, location, grade, and histological characteristics; TNM and Enneking stages; treatment details (surgical vs. non-surgical); and prognosis at the last follow-up visit (no evidence of disease, alive with disease, dead of disease, or dead of other causes). Patients who were registered less than 2 years from the study enrollment date were excluded. Data on 2651 patients with primary bone sarcoma were extracted from the database. Cases with insufficient data were excluded.

### Statistical analyses and study size

The primary endpoint was the occurrence of tumor-related deaths. The cancer survival time was defined as the period from the date of diagnosis to the tumor-related death. Patients without tumor-related deaths, or patients who died due to other causes, were censored at their last follow-up visit. The cancer survival rates for overall bone sarcoma (all types), osteosarcoma, chondrosarcoma, and Ewing sarcoma were calculated using the Kaplan-Meier method. Cox proportional hazards models were used to analyze the prognostic factors for cancer survival. The variables selected for the analysis were previously reported to be related to cancer survival [13–16]. Control variables for multivariate analysis were indicated by “reference”, including AYA, males, low grade, ≤8 cm, upper extremity, salvaged and negative. The level of significance was set at *P* < 0.05.

IBM SPSS version 19.0 software (IBM SPSS, Armonk, NY, USA) was used for all statistical analyses. The study size was dictated by the total number of patients with bone sarcoma in the BSTT database during the study period.

## Results

The study included 3457 patients with bone sarcoma (1930 male and 1527 female) who were registered in the BSTT database from 2006 to 2013. Table 1 shows characteristics of bone sarcomas in AYAs by age at diagnosis and relevant clinical factors. The frequency of bone sarcoma occurrence was highest in AYA patients, who accounted for a marked proportion of patients with each type of sarcoma. Except for this, no categories were notably more or less prevalent in the AYA patient groups when compared with the same categories in other age groups. The majority of AYA had osteosarcoma 631 (56.2%), while 198 (17.6%) had chondrosarcoma. Among children, osteosarcoma was the most common 405 (77.7%), while 92 (17.7%) had Ewing sarcoma. Chondrosarcoma was the most common among adults 376 (38.3%), while 278 (28.3%) had osteosarcoma. Finally, among elderly, chondrosarcoma was the most common 303 (36.5%), while 183 (22.0%) had osteosarcoma. The incidences of osteosarcoma and Ewing sarcoma decreased with age, while the incidences of chondrosarcoma increased with age.

**Table 1:** Characteristics of bone sarcomas in AYAs by age at diagnosis and relevant clinical factor

	Overall		AYA		Child		Adult		Elderly		*P* value
			(15-39 years)		(−14 years)		(40-64 years)		(65- years)		
	N	%	N	%	N	%	N	%	N	%	
Total	3457		1123	32.5%	521	15.1%	982	28.4%	831	24.0%	
Histologic subtype											< 0.001
Osteosarcoma	1497	43.3%	631	56.2%	405	77.7%	278	28.3%	183	22.0%	
Chondrosarcoma	885	25.6%	198	17.6%	8	1.5%	376	38.3%	303	36.5%	
Ewing’s sarcoma	260	7.5%	139	12.4%	92	17.7%	28	2.9%	1	0.1%	
Bone MFH	205	5.9%	22	2.0%	2	0.4%	82	8.4%	99	11.9%	
Chordoma	253	7.3%	16	1.4%	2	0.4%	88	9.0%	147	17.7%	
HG sarcoma(others)	214	6.2%	52	4.6%	4	0.8%	85	8.7%	73	8.8%	
LG sarcoma(others)	143	4.1%	65	5.8%	8	1.5%	45	4.6%	25	3.0%	
Sex											0.028
Male	1930	55.8%	656	58.4%	278	53.4%	561	57.1%	435	52.3%	
Female	1527	44.2%	467	41.6%	243	46.6%	421	42.9%	396	47.7%	
Tumor size (cm), mean [SD]	9.1 [4.9]		8.8 [4.5]		10.3 [4.8]		8.9 [5.1]		9.0 [5.1]		< 0.001
≤8 cm	1655	47.9%	538	47.9%	193	37.0%	510	51.9%	414	49.8%	
> 8 cm and ≤ 16 cm	1299	37.6%	432	38.5%	246	47.2%	322	32.8%	299	36.0%	
> 16 cm	243	7.0%	61	5.4%	50	9.6%	67	6.8%	65	7.8%	
Unknown	260	7.5%	92	8.2%	32	6.1%	83	8.5%	53	6.4%	
Tumor location											< 0.001
Upper extremity	349	10.1%	134	11.9%	40	7.7%	105	10.7%	70	8.4%	
Lower extremity	1689	48.9%	629	56.0%	395	75.8%	399	40.6%	266	32.0%	
Trunk	1276	36.9%	303	27.0%	72	13.8%	437	44.5%	464	55.8%	
Head and neck	35	1.0%	18	1.6%	2	0.4%	10	1.0%	5	0.6%	
Multiple disease	108	3.1%	39	3.5%	12	2.3%	31	3.2%	26	3.1%	
Surgery	2473	71.5%	868	77.3%	430	82.5%	713	72.6%	462	55.6%	< 0.001
Chemotherapy	1765	51.1%	769	68.5%	474	91.0%	374	38.1%	148	17.8%	< 0.001
Radiotherapy	724	20.9%	188	16.7%	81	15.5%	206	21.0%	249	30.0%	< 0.001

*SD* standard deviation, *AYA* adolescent and young adult, *MFH* malignant fibrous histiocytoma, *HG sarcoma* High grade sarcoma, *LG sarcoma* Low grade sarcoma

**Fig. 1:**
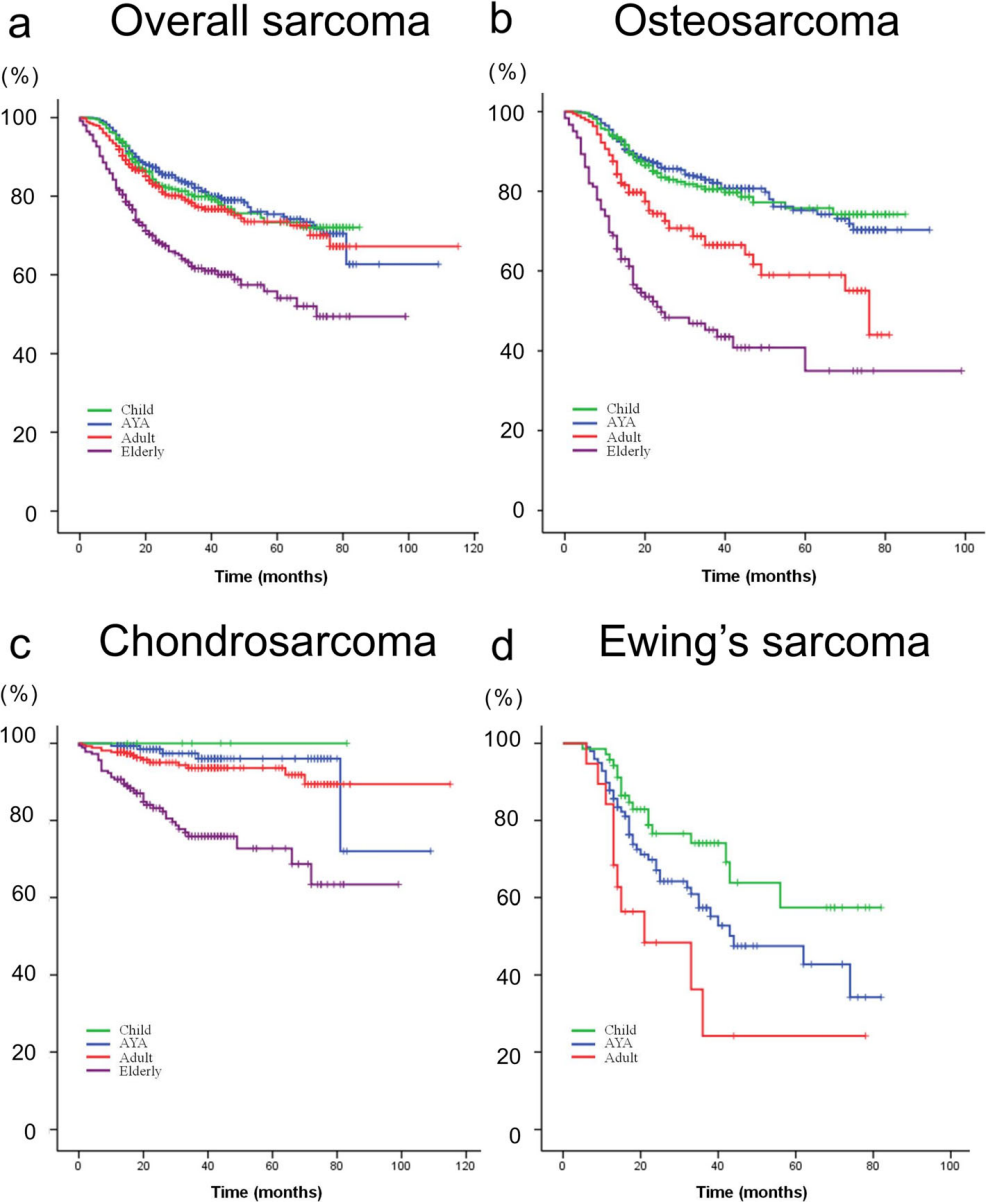
**a**-**d** Kaplan-Meier survival curves showing disease-specific survival for overall sarcoma (**a**), osteosarcoma (**b**), chondrosarcoma (**c**), and Ewing sarcoma (**d**), stratified by age. Child: ≤14 years, adolescent and young adult (AYA): 15–39 years, adult: 40–64 years, and elderly: ≥65 years. No elderly patients were included in **d** as no elderly patients were diagnosed with Ewing sarcoma

Figure 1a–d shows the cancer survival curves for patients with overall bone sarcoma (all types), as well as in those with osteosarcoma, chondrosarcoma, and Ewing sarcoma. There were no elderly patients with Ewing sarcoma. The cancer survival rate of AYA patients with osteosarcoma tended to be similar to that of children, but was better than those of adult and elderly patients. The cancer survival rate of AYA patients with chondrosarcoma tended to be similar to those of adults and was better than that of elderly patients. The cancer survival rates of children, AYA, and adult patients with Ewing sarcoma exhibited distinct tendencies, while the cancer survival rates of patients with Ewing sarcoma worsened with advancing age.

Table 2 shows the 5-year cancer survival statistics by age and sarcoma type. AYA patients with overall bone sarcoma did not exhibit worse cancer survival rates; however, the cancer survival was inversely correlated with age. The same tendencies were observed for each of osteosarcoma, chondrosarcoma, and Ewing sarcoma.

**Table 2:** Five-year survival statistics by age and sarcoma type

	All sarcomas		Osteosarcoma		Chondrosarcoma		Ewing sarcoma	
	N	5-year survival (%)	N	5-year survival (%)	N	5-year survival (%)	N	5-year survival (%)
Overall	2651	71.3%	1124	68.4%	603	88.0%	187	49.0%
Age at diagnosis								
AYA	912	75.3%	483	75.2%	150	96.0%	98	47.5%
Child	431	73.8%	327	75.7%	7	100.0%	70	57.5%
Adult	741	74.2%	192	59.0%	264	93.6%	19	24.2%
Elderly	567	58.5%	122	35.0%	182	72.7%	NA	

*AYA* adolescent and young adult

Table 3 shows univariate and multivariate analyses of prognostic factors of cancer survival by sarcoma type. Overall, the prognostic factors associated with poor cancer survival in patients with overall bone sarcoma were age ≥ 65 years (hazard ratio [HR]: 3.74; 95% confidence interval [CI]: 2.66–5.28; *P* < 0.001), high tumor grade (HR: 3.77; 95% CI: 1.93–7.37; *P* < 0.001), tumor size > 16 cm (HR: 2.20; 95% CI: 1.52–3.19; *P* < 0.001), and positive surgical margins (HR: 1.78; 95% CI: 1.21–2.62; *P* = 0.004) (Table 3).

**Table 3:** Univariate and multivariate analyses of prognostic factors of cancer survival by sarcoma type

	All sarcomas		Osteosarcoma		Chondrosarcoma		Ewing sarcoma	
	Univariate	Multivariate	Univariate	Multivariate	Univariate	Multivariate	Univariate	Multivariate
	HR (95% CI)	HR (95% CI)	HR (95% CI)	HR (95% CI)	HR (95% CI)	HR (95% CI)	HR (95% CI)	HR (95% CI)
Age at diagnosis								
AYA	Reference	Reference	Reference	Reference	Reference	Reference	Reference	Reference
Child	1.04 (0.79–1.36)	0.83 (0.59–1.18)	1.02 (0.73–1.42)	1.00 (0.70–1.43)			0.57 (0.33–1.00)	0.35 (0.15–0.83)
Adult	1.14 (0.92–1.43)	1.61 (1.16–2.24)	1.99 (1.42–2.78)	1.58 (1.11–2.24)	1.82 (0.66–5.02)	1.77 (0.63–4.94)	1.92 (0.98–3.74)	1.97 (0.69–5.65)
Elderly	1.99 (1.61–2.46)	3.74 (2.66–5.28)	4.35 (3.14–6.02)	3.26 (2.29–4.64)	7.38 (2.91–18.75)	6.13 (2.38–15.75)	NA	NA
Sex								
Male	Reference	Reference	Reference	Reference	Reference	Reference	Reference	Reference
Female	0.93 (0.79–1.10)	0.85 (0.68–1.06)	1.01 (0.80–1.29)	0.96 (0.75–1.23)	1.07 (0.64–1.78)	1.20 (0.70–2.06)	0.96 (0.60–1.55)	1.08 (0.53–2.19)
Histologic grade								
Low	Reference	Reference			Reference	Reference		
High	6.63 (4.71–9.34)	3.77 (1.93–7.37)			4.73 (2.75–8.14)	3.27 (1.84–5.83)		
Tumor size								
≤8 cm	Reference	Reference	Reference	Reference	Reference	Reference	Reference	Reference
> 8 cm and ≤ 16 cm	1.84 (1.53–2.21)	1.26 (0.99–1.62)	1.74 (1.32–2.30)	1.63 (1.23–2.16)	2.79 (1.55–5.02)	2.03 (1.12–3.70)	0.86 (0.51–1.44)	0.55 (0.25–1.23)
> 16 cm	2.92 (2.21–3.87)	2.20 (1.52–3.19)	2.65 (1.74–4.030)	2.84 (1.86–4.35)	4.81 (2.30–10.07)	3.06 (1.40–6.68)	3.00 (1.41–6.37)	2.39 (0.87–6.57)
Tumor location								
Upper extremity	Reference	Reference	Reference	Reference	Reference	Reference	Reference	Reference
Lower extremity	1.64 (1.14–2.37)	1.41 (0.94–2.12)	1.30 (0.79–2.14)	1.19 (0.72–1.98)	2.65 (0.78–9.04)	2.30 (0.66–8.03)	1.37 (0.52–3.61)	1.63 (0.51–5.22)
Trunk	2.48 (1.72–3.59)	1.43 (0.91–2.23)	4.43 (2.63–7.46)	2.64 (1.53–4.56)	4.40 (1.36–14.25)	3.62 (1.08–12.15)	1.01 (0.40–2.55)	1.19 (0.36–3.91)
Head and neck	1.93 (0.75–4.95)	0.82 (0.19–3.51)	1.35 (0.40–4.60)	1.73 (0.50–6.04)			1.78 (0.21–15.34)	5.16 (0.53–50.02)
Multiple disease	5.99 (3.68–9.74)	2.60 (1.17–5.78)						
Limb salvage								
Salvaged	Reference	Reference	Reference	Reference	Reference		Reference	
Amputated	2.15 (1.72–2.70)	2.98 (2.28–3.89)	2.47 (1.83–3.33)	2.80 (2.05–3.83)	1.56 (0.62–3.96)		2.01 (0.80–5.07)	
Surgical margin								
Negative (wide or marginal)	Reference	Reference	Reference		Reference		Reference	Reference
Positive (intralesional)	1.14 (0.81–1.60)	1.78 (1.21–2.62)	1.26 (0.52–3.07)		0.68 (0.26–1.76)		3.67 (1.60–8.40)	5.28 (1.90–14.62)

*AYA* adolescent and young adult, *HR* Hazard ratio, *CI* confidence interval

The results of univariate and multivariate analysis of prognostic factors for cancer survival in patients with osteosarcoma are shown in Table 3. Upon multivariate analysis, the negative prognostic factors included age 40–64 years (HR: 1.58; 95% CI: 1.11–2.24; *P* < 0.001), age ≥ 65 years (HR: 3.26; 95% CI: 2.29–4.64; *P* < 0.001), tumor size > 16 cm (HR: 2.84; 95% CI: 1.86–4.35; *P* < 0.001), and tumor location on the trunk (HR: 2.64; 95% CI: 1.53–4.56; *P* < 0.001) (Table 3). AYA patients had a similar HR to children and did not exhibit an increased risk of tumor-related death compared with the other age groups.

Likewise, the prognostic factors associated with poor cancer survival in patients with chondrosarcoma were age ≥ 65 years (HR: 6.13; 95% CI: 2.38–15.75; *P* < 0.001), tumor size > 16 cm (HR: 3.06; 95% CI: 1.40–6.68; *P* = 0.005), and tumor location on the trunk (HR: 3.62; 95% CI: 1.08–12.15; *P* = 0.038) (Table 3). Being in the AYA age group did not increase the risk of tumor-related deaths compared with the child and adult groups; furthermore, the risk of tumor-related deaths in AYA patients was lower than that in the elderly group.

Lastly, the sole prognostic factor associated with poor cancer survival in patients with Ewing sarcoma was a positive surgical margin (HR: 5.28; 95% CI: 1.90–14.62; *P* = 0.001). AYA patients had an increased risk of tumor-related deaths compared with children (HR: 0.35; 95% CI: 0.15–0.83; *P* = 0.016), but not with adults. None of the patients with Ewing sarcoma in our study were ≥ 65 years of age (Table 3).

## Discussion

There have been fewer improvements in the clinical outcomes of AYA patients with cancer than for children and older adults, possibly because fewer studies focus on patients in this age group. Our study revealed the outcomes of AYA patients with bone sarcomas. We found that the AYA age group was not an independent poor prognostic factor for bone sarcoma overall, or for osteosarcoma, chondrosarcoma, or Ewing sarcoma individually. This was in contrast to other cancers, such as those of the breast and colon [17]. The cancer survival rate in AYA patients with bone sarcoma was similar to that of children and adults, and was more favorable than that of elderly patients. However, there have been no significant improvements in the overall 5-year survival rates for patients with bone sarcoma over the past few decades, unlike other cancers [10]. It is possible that this finding is the same in Japan. There have been significant improvements in the overall 5-year relative survival rates for patients with other cancers because of established effective chemotherapy and molecular targeted drugs [18, 19]. It is possible that AYA patients do not stand out because there have been no improvements in the overall 5-year relative survival rates for other age groups.

In addition, insurance rates are significantly lower in AYA patients [20]. AYA cancer survivors without health insurance do not receive cancer-related medical care, while those with insurance do [21]. In Japan, every Japanese person belongs to the public medical insurance that bears 70–90% of the treatment costs. Japan has a national bail out system for officially acknowledged people in need, which covers almost 100% of the actual treatment costs. It is possible that cancer survival rates of AYA patients did not differ from patients in other age groups because patients of all ages received equal medical treatment. To our knowledge, this study is the first to investigate bone sarcomas based on age groups, including AYAs, and their clinical outcomes [22, 23].

Previous epidemiological analyses conducted in Australia and the United States showed that the cancer survival rates of AYA patients with osteosarcoma were significantly worse than those of children [10, 11]. However, our study showed that Japanese AYA patients with osteosarcoma had cancer survival rates that were statistically equivalent to those in children. The standard chemotherapy for osteosarcoma is methotrexate, doxorubicin, and cisplatin (MAP). Although the use of chemotherapy children and AYA patients was high, it was infrequently used in adult patients (data not shown). This likely explains why the cancer survival rates of AYA patients with osteosarcoma were better than those in adult and elderly patients. One other possible reason is that in Japan, one of the inclusion criteria for many clinical trials regarding osteosarcoma is patients aged ≤40 years [24–26]. AYA patients with osteosarcoma receive the same therapy as children. Tumor size is one of prognostic factors associated with poor survival among those with osteosarcoma [27]. In this study, there were few differences in the mean tumor size between AYA and adult patients (Additional file 1). Although children had the best osteosarcoma outcomes, the mean tumor size in children was the largest. For chondrosarcoma in particular, we found no other published studies with which to compare our results. The cancer survival rates of elderly patients with chondrosarcoma in our study were inferior to those of other age groups. One possible reason for this might be that elderly patients cannot be treated using surgery as a result of their advanced age. The proportions of patients who underwent surgery in the various age groups were as follows: children, 85.7%; AYA, 86.0%; adults, 87.1%; elderly, 73.1% (data not shown).

Tumor size is also a prognostic factor in Ewing sarcoma [28]. In this study, there were no significant differences in the mean tumor size between the age groups. One possible reason for this might be the distinct biological features of Ewing sarcoma in different age groups. It was reported that a gain in chromosome 1q and a loss in chromosome 16q were each associated with significantly worse outcomes; these mutations were more common in patients’ ≥15 years of age than in children [29]. Hence, the biology of Ewing sarcoma in AYA patients appears to be distinct from that in children [29]. In our study the frequency of Ewing sarcoma in AYA patients in Japan was lower than that in Australia and the United States [9, 11]. This is the reason why Caucasian populations are much more frequently affected, while there are low rates of the disease in East Asian and African populations [30].

The other independent risk factors for poor cancer survival in patients with bone sarcoma, as revealed in our study, are similar to those in previous studies of similar types of sarcoma. Consistent with our study, previous studies also reported that older age, large tumor size, high grade, and positive surgical margins were major factors that adversely influenced prognoses [28, 31–33].

Our study had several limitations. First, the BSTT Registry was computerized in 2006, and no long-term observations of over 10-years were possible. Second, there were many patients for whom functional outcomes were not recorded in the BSTT Registry; these would have been useful to evaluate. Third, AYA cancer survivors experience adverse effects on their quality of life that persist beyond cancer diagnosis and treatment, including issues with infertility, body image, difficulty establishing relationships, and many other aspects of physical and social functioning [18]. There are no data with which to evaluate such parameters in the BSTT Registry. Forth, due to the extremely low incidence rate, the number of children with chondrosarcoma and elderly with Ewing sarcoma was insufficient. However, despite these limitations, our findings provide detailed information on the epidemiology of bone sarcoma among AYAs in Japan.

## Conclusions

Our study is the first to provide data on the descriptive epidemiology and clinical outcomes of AYA patients with bone sarcomas using a nationwide, large-scale database. We found that, contrary to expectations, cancer survival rates of AYA patients with bone sarcomas were not inferior to those of other age groups in Japan.

## Additional file

Additional file 1: Tumor size by age and sarcoma type. (DOCX 23 kb)

## Abbreviations

AYA: Adolescent and young adult
BSTT: Bone and soft tissue tumor (registry)
CI: Confidence interval
HR: Hazard ratio
