# Supplementary material for: Descriptive epidemiology and outcomes of bone sarcomas in adolescent and young adult patients in Japan
Source: BMC Musculoskelet Disord. 2018 Aug 18;19:297. doi: 10.1186/s12891-018-2217-1 (PMC6098838; doi:10.1186/s12891-018-2217-1)
Supplement: Supplementary file 1 — Tumor size by age and sarcoma type. (DOCX 23 kb) [file 12891_2018_2217_MOESM1_ESM.docx]

| Appendix. Tumor size by age and sarcoma type | | |  |  |  |
| --- | --- | --- | --- | --- | --- |
|  | AYA | Child | Adult | Elderly | P value |
| **(cm), mean [SD]** | (15-39years) | (-14years) | (40-64years) | (65- years) |  |
| **All sarcomas** | 8.8 [4.5] | 10.3 [4.8] | 8.9 [5.1] | 9.0 [5.1] | <0.001 |
| **Osteosarcoma** | 9.5[4.2] | 10.7[5.0] | 10.0[5.1] | 10.2[4.7] | 0.003 |
| **Chondrosarcoma** | 7.6[4.7] | 9.7[3.2] | 8.1[5.4] | 9.4[6.1] | 0.042 |
| **Ewing's sarcoma** | 10.0[4.7] | 8.9[3.9] | 10.3[4/3] | NA | 0.237 |
| SD: standard deviation, AYA: adolescent and young adult. | | | |  |  |
